# An Examination of the Ethical and Legal Limits in Implementing “Traceback Testing” for Deceased Patients

**DOI:** 10.1017/jme.2023.23

**Published:** 2022

**Authors:** Jessica Martucci, Yolanda Prado, Alan F. Rope, Sheila Weinmann, Larissa White, Jamilyn Zepp, Nora B. Henrikson, Heather Spencer Feigelson, Jessica Ezzell Hunter, Sandra Soo-Jin Lee

**Affiliations:** 1: HISTORY AND SOCIOLOGY OF SCIENCE DEPARTMENT, UNIVERSITY OF PENNSYLVANIA, PHILADELPHIA, USA; 2: DEPARTMENT OF TRANSLATIONAL AND APPLIED GENOMICS, CENTER FOR HEALTH RESEARCH, KAISER PERMANENTE NORTHWEST, PORTLAND, OR, USA; 3: CENTER FOR HEALTH RESEARCH, KAISER PERMANENTE NORTHWEST; PORTLAND, OR AND GENOME MEDICAL; SOUTH SAN FRANCISCO, CA, USA; 4: INSTITUTE FOR HEALTH RESEARCH, KAISER PERMANENTE, DENVER CO, USA; 5: KAISER PERMANENTE WASHINGTON HEALTH RESEARCH INSTITUTE, SEATTLE WA, USA; 6: GENOMICS, ETHICS, AND TRANSLATIONAL RESEARCH PROGRAM, RTI INTERNATIONAL, RESEARCH TRIANGLE PARK, NC, USA; 7: DIVISION OF ETHICS, DEPARTMENT OF MEDICAL HUMANITIES AND ETHICS, COLUMBIA UNIVERSITY, NEW YORK, NY, USA

**Keywords:** Traceback Testing, Cascade Screening, Return of Results, *BRCA1*/*BRCA2*, Hereditary Breast and Ovarian Cancer, Cancer Syndromes

## Abstract

This paper examines the legal and ethical aspects of traceback testing, a process in which patients who have been previously diagnosed with ovarian cancer are identified and offered genetic testing so that their family members can be informed of their genetic risk and can also choose to undergo testing. Specifically, this analysis examines the ethical and legal limits in implementing traceback testing in cases when the patient is deceased and can no longer consent to genetic testing.

Recent estimates suggest that over 20% of ovarian cancers are linked to germline pathogenic variants (PV’s) associated with an increased cancer risk.^1^ PV’s in at least 11 different genes, including *BRCA1* and *BRCA2 (BRCA1/2)*, confer an increased risk for ovarian cancer.^2^ A recent study by Kurian *et al.* found that 14.5% of ovarian cancer patients tested positive for one of the known pathogenic variants currently associated with the disease.^3^ Despite the significant genetic component to this disease, studies estimate that fewer than 14% of *BRCA1/2* PV carriers are identified, with lower rates for PV carriers in the other genes linked to ovarian cancer risk.^4^ Increasing access to genetic risk information about ovarian cancer intersects with health justice and equity concerns, especially when it is used to reach underserved populations who are less likely to learn about their cancer risk due to barriers to access to healthcare.^5^ Identifying those at increased genetic risk for ovarian cancer as well as ensuring their at-risk biological relatives are informed and can access genetic testing continue to challenge the medical genetics community.

**Table 1:** Possibilities for Disclosure and their Legal, Ethical, and Pragmatic Implications

Disclosure to:	HIPAA	Ethics	Pragmatic
Deceased LAR	Requires that the deceased’s health information remains under the protection of a legally appointed representative for 50 years after their death.	Protects the deceased’s privacy. Limits the extent to which researchers can honor the right to know by contacting relatives.	Logistically complicated, costly, and difficult to identify the LAR.
Clinician-to-clinician	Health information can be disclosed for treatment purposes only.	Potentially infringes on the deceased’s privacy. Honors the relative’s right to know. Bypasses the relative’s right not to know.	Healthcare providers may not be particularly knowledgeable about the nature or meaning of the information provided. Locating a relative’s physician is logistically complicated, difficult, and time-consuming.
Public Health Authority	Health information can be disclosed to public health authorities if it poses an urgent risk to others.	Potentially infringes on the deceased’s privacy. Honors the relative’s right to know. May bypass the relative’s right not to know. Could address issues of access, equity and social justice by making this information available through county public health agencies. Unequal access to healthcare services could contribute to issues of access, equity, and social justice.	Genetic risk information may not rise to the level of urgency that is typically involved in public health tracking. Public health system in U.S. is not particularly robust or well-funded.

Traceback testing involves genetic testing in patients who have had a prior diagnosis of ovarian cancer to see if they are carriers of a pathogenic variant that poses a high risk to biological relatives. First described by Samimi *et al.* (2017) during a 2016 workshop convened by the National Cancer Institute at the National Institutes of Health, traceback testing offers a promising approach to increasing access to important genetic health information about ovarian cancer for family members. It begins with risk assessment of the patient and, in cases when the patient is still living, genetic counseling, before proceeding to genetic testing. If the patient is found to be a carrier of a PV, genetic counseling and testing can then be offered to family members through a process called cascade screening.^6^ Traceback testing can also be carried out through the genetic testing of stored biospecimens. Though less examined in the literature, this approach offers an additional opportunity for families to receive important genetic risk information even after their family member has died. Pathology departments routinely collect specimens during the course of regular clinical and oncological care. However, patients and their families are unlikely to know that these tissue samples exist or that they could be used in genetic testing that could offer potentially important information for their health and the health of their relatives.

In the pages that follow, we identify and examine the development of the relevant ethical and legal frameworks surrounding traceback testing and discuss the limitations of these for the specific case of traceback testing from stored pathology specimens after a patient is deceased. We argue that traceback testing, more generally, can serve as a means to greater health justice and equity, but in order to realize its full potential we must transcend traditional legal and ethical frameworks that fail to fully recognize the value of genetic health information beyond the bounds of a single individual. In doing so we highlight the need for a legal and ethical framework that acknowledges the extent to which genetic health information is relevant not only for individual patients, but for their biological relatives. Furthermore, we suggest that traceback testing demands new ways of conceptualizing the relationship between the ethics of the clinic and the lab, the deceased and the living, the patient in clinical care and the greater good in scientific research.

In preparation for our own traceback study among deceased patients with ovarian cancer, we examined the ethical and legal issues surrounding the recruitment of family members of deceased patients who had a diagnosis of ovarian cancer, but had not undergone genetic testing. Working with tissue samples of a deceased patient for the purposes of extracting genetic risk information to disclose to living family members raises unique legal and ethical questions. We have found little direct guidance in the existing literature, or in state and federal guidelines, to help us navigate a path through the specific legal and ethical circumstances raised by this work.^7^ In order to identify and address these adequately, here we analyze the complex evolution of ethical and legal approaches to two related subjects: cascade screening and return-of-results. Cascade screening is a systematic process of case finding that begins with the identification of an individual living with a disease-linked pathogenic variant, and then invites testing of their at-risk biological relatives.^8^ Traceback testing uses cascade screening techniques once an individual with a pathogenic variant has been identified. Return of individual research results is an issue that has emerged within the bioethics literature as more and more individualized health-relevant genetic data is created through genetic research. Scholars and researchers have increasingly called for reciprocity in genetic research so that participants benefit from studies through obtaining access to important genetic health information for themselves, and their family members.^9^ The ethical issues surrounding return of results intersect with those in traceback testing over issues of consent and disclosure of genetic information to patients and family members, particularly when the patient is deceased.

In the pages that follow, we identify and examine the development of the relevant ethical and legal frameworks surrounding traceback testing and discuss the limitations of these for the specific case of traceback testing from stored pathology specimens after a patient is deceased. We argue that traceback testing, more generally, can serve as a means to greater health justice and equity, but in order to realize its full potential we must transcend traditional legal and ethical frameworks that fail to fully recognize the value of genetic health information beyond the bounds of a single individual. In doing so we highlight the need for a legal and ethical framework that acknowledges the extent to which genetic health information is relevant not only for individual patients, but for their biological relatives. Furthermore, we suggest that traceback testing demands new ways of conceptualizing the relationship between the ethics of the clinic and the lab, the deceased and the living, the patient in clinical care and the greater good in scientific research.

To do this, we first examine the evolution of the ethical and legal landscape that shapes contemporary cascade screening methods. We then turn to a discussion of the ethical and legal issues raised by the return of genetic research results, paying particular attention to cases where the participant is deceased. Finally, we consider the legal aspects of consent and disclosure in these cases through a discussion of the Health Insurance Portability and Accountability Act of 1996 (HIPAA) Privacy Rule which protects health information (PHI) after a person’s death. We trace the available approaches to sharing identifiable genetic risk information with family members in clinical and research settings under the Privacy Rule, highlighting the limits and ethical concerns raised by each. Before closing, we return to a discussion of the ethical, legal, and practical questions that remain, highlighting the way these considerations have shaped, and ultimately limited, our study’s efforts to offer traceback testing to the living relatives of deceased patients.

## From the Emergence of Genetic Testing to Cascade Screening

Considering the history of genetic testing more generally is useful for understanding cascade screening’s potential for improving individual and public health outcomes as well as the legal and ethical frameworks that surround it. The utility of cascade screening relies not only on targeted genetic testing of populations with a known risk for a pathogenic variant, but on the ability to offer some sort of reasonable intervention once a variant is found. Some of the earliest genetic health screenings that offered an intervention were those in prenatal medicine, designed to provide parents with the choice to abort a fetus with significant genetic health issues.^10^ The development of a test for phenylketonuria (PKU) made it the first genetic disease for which screening (through the heel prick) appeared to offer a life-saving clinical intervention in the form of a low-phenylalanine diet.^11^ The potential impact of detecting the disease at birth led to the implementation of widespread public screening programs across the US. However, follow-up data began to highlight that this largescale screening approach also caused new problems, like overdiagnosis and false positives. It turned out that not all the children who tested positive in the screening actually required the special diet.^12^

The discovery of a genetic screening test for pathogenic variants linked to cystic fibrosis in the late 1980s is another important moment in this story.^13^ The CF test, however, also gave rise to a whole host of new ethical and practical questions for researchers, clinicians, and bioethicists to consider even as public expectations and enthusiasm for the medical technology grew.^14^ Excitement about CF screening waned, however, as feasibility studies showed that the costs and ethical complexity of another widespread genetic screening program might outweigh the benefits.^15^ Researchers, for example, noted the increase in unnecessary anxiety that widespread carrier testing for CF caused, as well as the high financial cost and logistical complexity of such a mass screening program.^16^

Given these concerns with mass genetic screening programs, geneticists began to experiment with the idea of cascade testing — “starting with individuals with a family history and their spouses, then offering to test the relatives of all those who are positive.”^17^ By targeting specific populations with a known risk for carrying a pathogenic variant, cascade screening offered the chance to more fully integrate genetic testing into healthcare, while lowering the costs as well as the risks of overdiagnosis and false-positives that broader screening programs generated.^18^ By the end of the twentieth century, cascade screening for familial hypercholesterolemia (FH), a genetic condition that leads to high cholesterol and the potential for heart attacks, even at a relatively young age, emerged as the next big hope in genetic medicine.^19^

## Understanding the Ethical Landscape of Cascade Screening

Years of studying public responses to screening programs, by the early 2000s, helped illuminate key ethical issues that arose in tandem with the development of these cascade screening efforts. Studies into the psychological, economic, public health, and social impacts and limits of cascade screening sought to understand both what the utility of cascade screening was as well as how patients and their families viewed genetic health information and the processes surrounding it.^20^ Researchers and ethicists initially asked many practical questions like: Should cascade screening be adopted as a public health tool? ^21^ Under what circumstances and for what goals?^22^ Would knowing one’s genetic status for diseases like Huntington’s disease, CF, hereditary breast and ovarian cancer, and FH, change individual behavior and lead to better health outcomes?^23^ With the meteoric expansion of genetic research and the emergence of precision medicine during this era, more refined questions about how to collect and use genetic health information in the clinic also emerged. Researchers began to ask: What kind of genetic information should be considered clinically useful for individuals? What information is actually welcomed by individuals?^24^ How should family members be contacted and confronted with the opportunity to undergo genetic testing?^25^ And how might the results of this increased testing shape lives and health outcomes individually and at the population level?^26^ These considerations only became more relevant as the scale and depth of genetic information available expanded. Other questions surfaced, too, about the rights of individuals “not to know”^27^ their genetic status. These discussions about unwelcome knowledge cited examples in which patients who underwent genetic testing learned information that challenged their identity or kinship ties,^28^ as well as situations in which people became overwhelmed with anxiety, failed to act on the information altogether, or regretted or resisted learning their status.^29^

Critical work also pointed out that data produced through genetic testing could not always adequately capture the true risk of disease based solely on a genetic test since not all pathogenic variants are known and others, while present, may not always be expressed. The overuse of testing for conditions that are either very common or exceedingly rare also have limited utility. Individuals may be found to carry variants that have no direct clinical utility, like those in the MTHFR gene which impacts the production of an enzyme. Such variants are extremely common but carry only vague implications for a person’s health. In extremely rare disorders, risk information from genetic screening is difficult to parse. Furthermore, identifying pathogenic variants through genetic testing does not directly dictate an individual’s access to healthcare, choices, or health outcomes. ^30^ The recognition that there could be substantial negative impacts on individuals from exposure to genetic health information has led to the creation of guidelines and standards for offering genetic tests and screening. Organizations like the American College of Obstetrics and Gynecology and the American College of Medical Genetics and Genomics, for example, have supplemented an imperfect net of Federal regulations that have been governing genetic tests since the passage of the 1988 Clinical Laboratory Improvement Amendments.^31^ These patchwork efforts recognize that genetic health information, while often beneficial, can also have negative consequences.

The protection of genetic privacy and the uses of patient information, then, have also surfaced in these discussions about the potential harms of genetic testing, more generally.^32^ Scholars have raised concerns about the possibility of discrimination in the workplace and by insurance companies who might use genetic health information to deny or terminate insurance coverage and benefits, declare pre-existing conditions, and reject claims. These anxieties have helped to drive calls for the protection of genetic privacy. Eventually, these discussions helped inform the creation and passage of specific legislation in the form of the Genetic Information Nondiscrimination Act (GINA, 2009), meant to prohibit this genetic discrimination, particularly in employment and health insurance. This sensitivity to genetic privacy also influenced the 2013 revision of HIPAA.^33^

Cascade screening as a health tool has not emerged *de novo*. It has instead grown up within a broad and complex legal and ethical landscape that has been evolving since the 1970s, contextualized by a history steeped in the debates surrounding reproductive and prenatal medicine, health insurance, employment discrimination, and privacy. The majority of the legal and ethical discussions that have shaped this landscape for cascade screening over the past two decades have thus been heavily shaped by the weight of arguments rooted in concern for privacy and individual autonomy.^34^ Yet, in the case of cascade screening, the entire process is designed to look beyond the immediate interests of the individual, to include those of a “cascade” of biological relatives. Empirical studies on participant and family attitudes towards various aspects of the cascade testing process conducted over the past decade help illuminate the need for an ethical framework that gives greater weight to the rights of biological relatives when it comes to genetic health information.

## The “Right to Know”

Cascade screening can be a cost-effective and helpful tool in treating gene-linked diseases, but its implementation in the US has remained limited.^35^ Stakeholder studies conducted in European countries, however, have shown that family members conceptualize access to genetic risk information as a right.^36^ In the Netherlands a survey of patients (*n=379*) and healthcare professionals (*n=*1000) found that the majority of all respondents named a “right to know” for family members, and considered it to be a “dominant issue,” in determining the extent to which healthcare providers should be involved in contacting relatives about their genetic risk.^37^ The right to know is related to concerns about the “right *not* to know,” which has long played a role in conversations about screening of all sorts. The right not to know is often accompanied by discussions about avoiding psychological harm and respecting individual choice, autonomy, and privacy. It asks: Is it ethical, fair, or just to burden someone with information they did not seek out on their own? As preventive treatments for genetic diseases become more available, however, the ethics of this equation appear to be shifting^38^

Born out of the literature on incidental and secondary findings from genetic tests, the discussion of the “right to know,” has become increasingly salient in light of the possibilities of expanded genetic testing abilities.^39^ In the case of genetic health information, it asks: Is it fair to deprive a person of access to genetic risk information that they would not otherwise know about? The right to know has received less attention in the US, although today the American College of Medical Genetics recommends the return of results from genetic research for conditions they have deemed actionable.^40^ Perhaps the idea has been slow to gain traction in the U.S. because the right to know can conflict with longstanding cultural concerns for the protection of individual autonomy and privacy. The right to know, however, can be strengthened when seen in terms of equity and justice. We know, for example, that in the U.S., Black ovarian cancer patients are less likely to obtain genetic testing than white patients.^41^ Although the issue of underrepresentation in genetic research more generally remains a significant barrier, giving greater weight to the right to know may help reach populations that are disproportionately under-screened.

When it comes to sharing genetic risk information with individuals and family members, the right to know is also supported by the “duty to warn,” or the “duty of easy rescue,” two additional and related ethical considerations that have wound their way into genetic testing discussions. The duty to warn focuses on the ethical obligations faced by a clinician or researcher to warn “third parties about the threats of a dangerous patient”^42^ but also extends to informing “family or third parties of their patients’ infectious disease status or genetic risks when an actionable intervention against the threat exists.”^43^ Although there are a handful of court cases in which the duty to warn has withstood medical breeches of individual privacy for the benefit of family or others in harm’s way, these have been limited to extreme scenarios in which an individual or their health information poses an immediate threat to the welfare of others. These instances are limited because embracing the duty to warn can often mean breaking HIPAA’s Privacy Rule.^44^ Bioethics scholars have argued persuasively that the duty to warn must be balanced carefully against privacy concerns and an individual’s right not to know. They point out that it alone cannot be used to justify the return of genetic results to family members without the patient’s consent.^45^

The “duty of easy rescue” may also support the “right to know.” Generally, it suggests “if someone can prevent a serious harm to another person at minimal cost to herself, then she has a moral duty to do so.”^46^ Some have argued that this duty extends beyond clinicians and researchers to impact all participants in a health/research system. If we acknowledged the duty to easy rescue in the context of genetic health information, then genetic risk information might be more easily shared with the biological relatives of individuals who test positive for certain pathogenic variants. Some have even claimed that the duty to easy rescue supports the use of genetic health data from stored tissue specimens “for the benefit of society,” even without prior consent.^47^ Consensus seems to have emerged, however, around the argument that broad consent *is* required for the use of biospecimens, and so the application of the duty to rescue as a justification for sharing individuals’ genetic health information remains weak and is not particularly useful for the case of our traceback study.^48^

In the case of cascade screening in traceback testing, the family members’ right to know remains in tension with the privacy and autonomy rights of the patient. For this reason, the patient’s consent has remained essential for ethically and legally sharing genetic risk information with family members. This conflict between the rights of the patient vs. the family is most visible in the discussion about *how* genetic health information should be disseminated to biological relatives, and by whom, particularly in cases when the patient has died without leaving a clear statement of consent.

## Indirect vs. Direct Contact

By the early 2000s, the practical and ethical validity of cascade screening had been firmly articulated. Many felt strongly that cascade screening could be more widely implemented across a broader spectrum of diseases and disease risks, within certain contested ethical parameters, and that doing so could save lives.^49^ One of the details that researchers and ethicists continued to explore during this era was how best to get the family members of impacted patients to participate in cascade screening. Afterall, cascade screening can only be effective if biological relatives act on the information they receive about their risk by getting tested, themselves. These conversations focused almost exclusively on a scenario in which a patient was living, but today they provide an important ethical scaffolding for thinking about how to approach a traceback process in which the patient is deceased.

In the case of cascade screening in traceback testing, the family members’ right to know remains in tension with the privacy and autonomy rights of the patient. For this reason, the patient’s consent has remained essential for ethically and legally sharing genetic risk information with family members. This conflict between the rights of the patient vs. the family is most visible in the discussion about *how* genetic health information should be disseminated to biological relatives, and by whom, particularly in cases when the patient has died without leaving a clear statement of consent.

In “indirect contact,” a living patient is left to discuss information about their genetic health status and cascade screening options with their family on their own terms. Indirect contact is often seen as a good way to balance respect for the patient’s right to autonomy and privacy with beneficence and respect for the right to know of their relatives.^50^ Clinicians and researchers may discuss contacting family with the patient, but ultimately must leave it in their patient’s hands to decide how to proceed. In a “direct contact” approach, clinicians reach out to family members directly.^51^ There are significant benefits to direct contact, including the mounting evidence that the uptake of testing is higher when clinicians contact family members directly. Furthermore, empirical work has suggested that patients may actually prefer direct contact, especially when it is preceded by the patient obtaining verbal consent from their family members prior to the outreach. ^52^ This kind of approach highlights a middle-ground for recruitment into cascade screening that again aims to both respect the autonomy of the patient and the family members’ right to know.

Advocates of direct contact have generally focused on whether testing for a particular genetic variant will make a significant difference in reducing mortality or increasing wellbeing. In the case of familial hypercholesterolemia, for example, direct-contact advocates have often argued that the principle of beneficence and the obligation to respect family members’ wellbeing and autonomous decision-making ought to outweigh the patient’s right to privacy.^53^ Studies in the disclosure of pathogenic variants in *BRCA1*/2 have suggested, furthermore, that despite the increased burdens of guilt and worry over their health and the health of their relatives, that positive test results do prompt family members to engage in more active management and mitigation of their cancer risk — suggesting that the direct disclosure of genetic risk information may yield significant benefits for hereditary cancers, as well.^54^ Additionally, some have highlighted that the sense of duty and obligation that a patient may feel to discuss their genetic results with family can cause psychological harm, harm that could be mitigated through a direct-contact approach.^55^

Over the past two decades, researchers have produced a substantial body of empirical work to put different versions of these contact methods to the test. In the case of cascade screening in hereditary breast and ovarian cancer, a consensus has emerged around the direct approach because it yields a higher response from relatives. Still, the literature has continued to maintain the importance of securing the patient’s participation in the process of contact in some form whenever possible, acknowledging that familial relationships, privacy, and individual autonomy are best respected when the patient plays a role in communicating with their family.^56^ This hybrid, or “two-step” approach to direct contact has garnered significant support among clinicians and ethicists alike, many of whom approve of the balance that it strikes between the autonomy and privacy rights of the patient and the rights of their family members to know, and not know, as well as its higher success rate for enrollment.^57^

The success of this approach is exemplified by the practices of the South Australian Familial Cancer Service. In 2006, the authors’ published on their experiences with indirect vs. direct contact and helped to empirically demonstrate the benefits associated with disclosure of cancer risk information to relatives. Before undergoing genetic screening, participants were asked if they would assist the clinic in contacting their relatives if a pathogenic variant was found. If a participant tested positive for one of the variants in question, they were asked to provide contact information for their relatives and the clinic sent them a letter. On average, they saw almost twice as much uptake of genetic testing among family members in the direct contact vs. indirect contact cohorts. They also reported that direct contact with family members about genetic risk could reduce the stress and burden upon the patient to talk to their relatives. Finally, they found that direct contact minimized the undue pressure relatives might feel to pursue testing when confronted by a family member compared to the clinic team.^58^

Understanding the evolution of the ethics surrounding cascade testing is critical for thinking about the ethics of traceback testing. Both the “the right to know” and the groundwork on direct contact that have been established in the cascade screening discussions are vitally important because together they establish a path towards providing genetic risk information to families, even in cases when the patient is deceased. This discussion, however, is no longer confined to clinical settings. Over the past decade, researchers, participants, and ethicists have begun to raise similar discussions about the ethics surrounding genetic health data produced in the context of scientific research.

## Disclosure in Research to Participants and Their Families

In the 2010s, the conversation around the ethics of sharing genetic information and cascade screening shifted beyond the clinical realm and intersected with ongoing discussions about the return of results to participants in scientific research. Scholars began to point out that, unlike patients in the clinical setting, participants in genetic research may unwittingly expose themselves to the production of genetic information that they never intended to actively seek out, raising additional concerns about personal autonomy, the right not to know, and informed consent. As the line between clinical medicine and research blurred in genomics, however, researchers and ethicists sought to establish guidelines for how to return important health results to living research participants in an ethical way.^59^ Just as with cascade testing, many have argued that these concerns naturally extend to the family members of affected research participants, as well. In 2010, for example, Boddington wrote:

“Research in genomics creates challenges for the historically dominant approach to medical research ethics on at least 2 fronts. First, it generates data that has potential implications not just for the individual participant but also for biological relatives…Second, it tends to raise questions that concern harms that relate to information generated rather than physical harms. Concerns raised focus on issues such as privacy, confidentiality and rights to information. These concerns can then extend to relatives.”^60^

The ethical obligation of researchers towards participants has historically been framed as an exclusive relationship, in which obligations from researchers to participants generally cover respect for a participant’s autonomy, the minimization of harm, and the maximization of benefit.^61^ Within this framework, consensus eventually began to emerge around the argument that researchers do have an obligation to make clinically-relevant information generated by their work available to individual research participants,^62^ despite concerns that it might fuel therapeutic misconception — or the misinterpretation of research as clinical care.^63^ However, there has been far less consensus on whether or not the researchers’ obligation to a participant also extends to their family members, an issue that is particularly relevant if the participant dies. As Black and McClellan noted in 2011, “At stake here is a balance between the privacy rights of the research participant and the ‘right to know,’ not quite an established right of the participant’s family.”^64^

### HIPAA and Return of Results

Given the new possibilities raised by the intersection of the ethics behind return of results and cascade screening, bioethicists have sought to carve a path forward that balances what are often understood to be the competing interests of the research participant, the researchers, and the family members when the participant is deceased. When it comes to the question of direct disclosure of research results to family members, however, there is little to legally support this in the U.S. context. The weight given to patient autonomy and privacy in U.S. law is protected in Federal patient privacy legislation (HIPAA) as well as in state laws around the country. HIPAA’s Privacy Rule stipulates that patients must give their consent for their health information to be shared. The reach of HIPAA extends beyond the end of life by 50 years, requiring the appointment of a personal representative who can “stand in the shoes” of the decedent in any matters regarding protected health information.^65^

Under HIPAA, a personal representative is the only person who may authorize disclosure of the decedent’s protected health information, including genetic information, even if doing so might provide a significant health benefit to living family members. However, *who* a personal representative is and how they are appointed can vary from state to state, with each following different rules and unique orders of kinship. Because HIPAA covers information like birth dates, gender, contact, and emergency contact information, the barriers to tracking down a personal representative can be a formidable logistical obstacle that can stand in the way of researchers wishing to disclose information to family members when a participant is deceased.

The information on personal representatives is generally kept by local probate courts and county records’ offices and is not typically found in a patient’s medical record. Since storage of this information is up to each individual county, tracking down information about a deceased patient’s personal representatives could (rarely) be as simple as an internet search or it could require visiting and requesting a patient’s records in person. Even when records are kept online, it varies widely how frequently the records are updated. Often there can be fees associated with records requests to cover the administrative costs of locating and providing copies. As a result of these formidable legal and logistical barriers to locating and contacting the personal representatives and family members of deceased patients, researchers must grapple with the question of what to do when the research participant is deceased, and their family stands to benefit from access to important health information.^66^

### The Ethics of Disclosure After Death

At the beginning of the last decade, legal scholar and bioethicist Anne-Marie Tassé published what remains the most in-depth and comprehensive ethical analysis of “whether or not disclosure of [research] results to family members could be ethically acceptable,” when a patient is deceased.^67^ From a principalist perspective, Tassé argues that autonomy for both the patient and the relatives must be respected. Beneficence, however, calls attention to the fact that disclosure “will bring no benefit to the deceased” but “is likely to bring a significant benefit to the living family members.” Tassé suggests that “dead persons cannot be harmed,” and that any harm that might come to the family members through disclosure would be outweighed by the potential harm to their health and lives caused by non-disclosure. Tassé also calls attention to the principle of justice which “demands the fair distribution of benefits, risks, and costs.” It is justice, according to Tassé, that tips the balance in favor of returning results because “it allows for a better translation of individual research results into clinical practice” and distributes the benefits of that research to those who stand to benefit from it more directly.^68^ Furthermore, from a consequentialist perspective, Tassé argues that the “confidentiality and privacy requirements could be outweighed when the disclosure … is likely to cause the greatest good for the greatest number.” Tassé advises that there must still be a careful examination of six elements that must be balanced in order to “determine the ethics of the post-mortem return of research results,” and so each kind of situation must be examined in order to determine the most ethical course of action. These include: The global impact of the return of deceased individuals results on research in general; the sensitivity of research results; the time elapsed since the death of the research participant; the deceased’s wishes, memory and/or reputation; the family members’ wishes to receive (or not) the research results; and the relevance of the research results for the health of the living relatives.^69^ As Tassé’s work highlights, there are multiple existing ethical frameworks that allow for the return of genetic test results to family members when the patient is deceased. Furthermore, a growing body of empirical evidence suggests that the public generally favors sharing genetic information with family members after death, even if researchers and IRB committees show a greater reticence to do so.^70^

The return of genetic information to family members of a deceased patient or participant could confer significant benefits through a traceback program that can provide actionable risk information to relatives. The persistent conflict between the culturally and legally privileged privacy rights of the deceased vs. the less well-defined “right to know” of their living biological relatives, however, remains unresolved and has hindered efforts to develop an ethics of disclosure.^71^ Within discussions where disclosure of genetic risk for the benefit of biological relatives has been pursued, scholars have framed their discussion around the ethics of “active” (disclosure initiated by the researchers) vs. “passive” (initiated by the relatives) disclosure practices.

### Active vs. Passive Disclosure

In 2012, Chan *et al*. detailed a process by which whole-exome genome sequencing data had been disclosed to family members of deceased research participants. Through the example of a few cases, Chan *et al*. concluded that “certain kinds of results should be disclosed,” and that “absent specific directions from the research participant, the literature suggests that there is a presumption in favor of releasing genetic test results that are relevant to the health of the patient’s relatives.”^72^ Chan *et al*. also argued for a process of “passive disclosure” to be implemented in situations of whole exome and whole genome sequencing. By passive disclosure, they meant “family members must make the request” for the deceased participant’s information themselves and should not be approached by the research team. A policy of passive disclosure, they argued, would limit disclosure only to those families who sought to know the information, thus minimizing harm and maximizing benefit.^73^

Responses to the Chan *et al*. publication were mixed, with some supporting their passive approach to familial disclosure, while others advocated for a more active approach. Those in the latter camp argued that “the obligation to return research findings should be explicitly limited to well-validated information of high clinical urgency, and — on account of that urgency — that such information should be actively, and directly, disclosed to all at-risk family members.”^74^ Others pointed out that these same questions also applied to the vast stores of tissue and pathology samples stored in biobanks, raising a host of additional possibilities.^75^ As scholars in this camp noted, the whole point of biobanking is to store material for future research, “including testing with methods that are not currently developed.”^76^

As new techniques become available and new health-relevant data is extracted from these stored samples, the likelihood of dealing with the question of whether and how to contact a deceased patient’s relatives appears as though it will continue to grow.^77^ However, in their closing, the authors noted that they were “not discussing the *legality* of disclosure,” and that in their particular case neither the Common Rule for human subjects protection (45 CFR 46) nor HIPAA applied because the Common Rule does not cover deceased participants and the NIH is not a “covered entity,” subject to HIPAA. This side remark, however, turns out to be incredibly significant, a point that Rothstein highlighted in his commentary in response to the Chan *et al*. paper. Rothstein stated that “in the absence of a prior authorization by the research participant or the participant’s legally authorized representative, disclosing the results of research to the family members of a deceased participant is unlawful under the HIPAA Privacy Rule.” In other words, ethical nuances aside, the cases discussed in Chan *et al*. were highly unique because “the research was undertaken at the NIH, which is not a covered entity under the HIPAA Privacy Rule.” Rothstein rightly points out that “if the research had been conducted at a typical academic medical center or institution, then the HIPAA Privacy Rule would prohibit such disclosures.”^78^

Although compelling, the implications of this argument ought to give us pause. As Rothstein points out, the difference between Chan *et al*.’s work, as they describe it, and others pursuing similar research is HIPAA, a law passed essentially to protect patient’s private health information from becoming fodder for health insurance companies to deny coverage. That HIPAA has become an impediment to asking important questions, like the ones that Chan *et al*. have examined, is perhaps an unforeseen consequence, one that may force us to reconsider how it is implemented. HIPAA’s Privacy Rule serves as a significant obstacle for exploring the potential health impact of testing stored pathology samples. In the months and years following Chan *et al*.’s publication, consensus on the return of results to *participants* continued to grow, even as alternate views persisted.^79^ Some expressed worry that if return of results were to become more routinized, it would become an obligation that could overburden the research enterprise.^80^

Researchers continue to struggle over how to make sense of the imbalance created by the legal weight bestowed upon the privacy of the deceased against the moral and ethical pull of beneficence and justice principles that favor the right to know and the disclosure of genetic information to at-risk family members. While passive disclosure of genetic information to a deceased participant’s personal representative is understood to fall within the bounds of HIPAA, few family members are in a position to know either that it is within their rights to request such information or that it exists at all. Given that so little empirical evidence has been gathered in this area, it is unclear how many family members would even know that a deceased relative has left behind a biological sample that could carry important genetic health information or how to go about requesting access to it. In short, passive disclosure may fit the legal constraints of the Privacy Rule, but it does little to address the growing importance of the right to know, that is rooted in ethical claims of beneficence and justice.^81^

## Discussion: Seeking Practical Solutions

More recently, scholars who favor familial disclosure of genetic results have begun to seek alternatives that attempt to resolve the legal impossibility in the U.S. of direct disclosure of a deceased’s genetic information to living relatives, while still taking a more active approach than passive disclosure allows. So far, these possibilities include: (1) Disclosure to the deceased’s legally appointed representative (LAR;);^82^ (2) Disclosure to a relative through the relative’s physician for the purposes of their own treatment;^83^ and, as suggested by Henrikson *et al*., (3) Disclosure to a public health authority.^84^ Of these three options, however, little empirical evidence has been gathered as to how these would work in practice, what it would take to implement these efforts, and what the ethical implications are of these possibilities.

### Disclosure to the Deceased’s LAR

In our own work, we have opted to pursue the first of these three options, identifying and contacting the deceased’s LAR in order to request consent to conduct genetic testing on the deceased patient’s sample and to release that information to the patient’s living relatives. This is a challenging path. From our preliminary investigation, most individuals do not submit information on the executor of their will to their healthcare providers and the majority of the electronic medical records’ searches that we have conducted in the Kaiser Permanente Colorado and Northwest systems lack this information. Because many of the individuals whom we are interested in testing for ovarian cancer markers are deceased, the path towards identifying their personal representative is murky at best. Conservative interpretations of HIPAA’s privacy rule prevent reaching out to family members listed in the deceased’s electronic medical record to inquire about the identity of a personal representative, raising the question of the logistical possibility of following HIPAA’s guidelines and doing traceback testing for deceased patients at all. The question of *how one identifies* the personal representative in the case of a deceased patient when HIPAA prevents contacting anyone *but* the personal representative is one that has not been addressed in the literature, possibly because it is unresolvable. The possibility of contacting local county probate records’ offices to track down personal representative information is a complicated feat at the best of times and adds a costly burden to the research enterprise.

### Disclosure to a Clinician

The second option allowed under HIPAA would require researchers to reach out to the healthcare provider of a deceased patient’s family member. According to the Privacy Rule, a doctor can “disclose protected health information about a patient to another healthcare provider for the purpose of treating another patient (e.g., to assist the other healthcare provider with treating a family member of the doctor’s patient).”^85^ Although this falls explicitly under the rules laid out by HIPAA, this option raises concerning ethical and pragmatic issues. Sobel *et al*. (2020), for example, has made the ethical argument that results may only be provided directly to healthcare providers “with the participant’s consent.” They contend that “research results should not be dumped on unsuspecting healthcare providers, particularly if they are not familiar with the types of results provided.”^86^ Practically speaking, the logistics of releasing genetic health information from a deceased patient to a family member’s healthcare provider is far easier said than done. It seems unlikely that most family members would have healthcare providers within the same regional healthcare network, making it almost impossible to know *who* the relatives’ providers are. As expressed by Sobel *et al*., the ethics of “cold-calling” a family member’s provider and burdening them with information they themselves may not be equipped to address adequately seems dubious, at best, and practically seems unlikely to meet with much success.

### Disclosure to a Public Health Authority

Finally, the third option, disclosure to a public health authority outlined by Henrikson, *et al*., seems in many ways the most ideal.^87^ In this option, a clinician would report a patient’s genetic results to a public health authority, who would then assume responsibility for contacting relatives. This system would operate under existing public health systems, similar to the way some dangerous contagions are tracked and addressed, like tuberculosis, rabies, or more recently, COVID-19. Framing traceback testing in terms of public health ethics emphasizes the possibilities of equity and social justice and lays out a well-established path — primarily with infectious disease — for using individual health information to benefit a larger population. Genetic diseases, however, don’t fit easily under the purview and abilities of existing public health infrastructure. This is an area for further study. While it seems plausible, it remains currently untenable for genetic diseases, which are not contagious and do not generally pose eminent harm, within the existing institutional and government structures that make up the U.S. public health system.

Each of these three options, the LAR, the clinician-to-clinician, and the public health routes, provide substantial logistical obstacles and raise their own set of ethical concerns. In the case of the LAR path, we have encountered significant hurdles in trying to identify a deceased patient’s personal representative from their existing medical records. Even for those who have a listed LAR, the path towards contact and obtaining consent is riddled with awkwardness that may prove insurmountable. For example, if we may not release any PHI about a patient until we have determined the identity and authority of their LAR to act on their behalf, then how can our research team adequately explain to them why we are contacting them and requesting sensitive identity information about a deceased loved one? As our empirical work unfolds, we will undoubtedly find out just how difficult these sensitive conversations will be to navigate.

In the case of the clinician-to-clinician route, the questions of ethics and respect loom large — the practice of clinicians exchanging protected health information about a patient without their knowledge smacks of an earlier era when medical paternalism kept patients in the dark about “difficult” diagnoses, like cancer. Although the information would be exchanged with the intent of eventually disclosing it to the patient, this pathway comes dangerously close to subverting the trust between a patient and their doctor by reducing a patient’s autonomy to *not know*. Finally, while the public health option sounds promising in theory, the lack of an equitable health system in which the information about health risk would accompany adequate access and care poses a significant justice issue, challenges the patient’s right not to know, and expands the surveillance authority of public health far beyond its existing boundaries of contagious diseases, raising contentious political questions about the relationship between the state and its citizens as well as troubling possibilities for discrimination based on genetic risk.

### A Fourth Option? — Learning Healthcare Systems

Given the limitations of the existing possibilities, we are left to the time-consuming and limiting option of searching for personal representative information in the medical records of deceased patients. The onerous and restrictive context surrounding disclosure of health information that has been created by HIPAA, however, begs for re-evaluation in this era of translational medicine. This experience has prompted us to consider new frameworks that, in the future, would allow efforts like traceback testing to move forward with disclosure of genetic results to at-risk family members in the case of deceased patients, offering increased access and equity to important genetic health information.

One model we have begun to think critically about is that put forth by those who advocate for a learning health care system (LHCS) approach to clinical care and research. Proponents of LHCS have argued that the existing bioethical frameworks that currently structure what is possible in research and clinical care have been built upon rigid and increasingly antiquated distinctions between the research and clinical realms. Rather than try to resolve this conflict through patchwork approaches, Faden and colleagues suggest that our contemporary health system requires a new ethical framing altogether, one in which it is not only acceptable but “essential to integrate research and practice.”^88^ They argue that patients in the LHCS have an ethical obligation to “contribute to learning,” a contribution that they explicitly state can and should “extend to family members, loved ones, and surrogates of patients” in order to provide the most efficient, affordable, fair, and high quality health care.^89^ The ethical framework behind LHCS as outlined by Faden *et al*. stresses a conception of a reciprocal obligation through which LHCS clinicians, researchers, and patients share responsibilities. Consideration of the LHCS framework suggests the important role of healthcare organizations as moral agents in addressing concerns over privacy and the potential benefit of disclosure of information to family members. Reciprocity in the LHCS advocates that organizations should create pathways for disclosure to optimize the welfare of their members.

We suggest that traceback testing for the identification of genetic health risks in living family members who may be unaware of their genetic risk is one example of how a shift away from the bifurcated ethics of the past, devised on the basis of a stark methodological and theoretical divide between research and clinical medicine, and towards a more integrated ethical framework may maximize the benefits of research for participants and patients, their relatives, and the population more generally. As more genetic health risks become identified and identifiable in individuals, the potential benefits of traceback testing will grow and widen. Targeted screening through traceback methods can recognize the right to know of biological relatives, focus resources on those with a familial genetic risk, reduce the harms of overdiagnosis and false positives that can result from mass screening programs, and steer us away from the generation of an overwhelming amount of decontextualized genetic health information. In order to fully explore the possibilities in traceback testing, however, we need to find a way to move beyond the longstanding ethical dichotomy that pits an individual’s autonomy and privacy rights against issues of justice and the family’s right to know.

The LHCS offers one possibility for imagining a new ethical framework in which patients and research participants share an obligation to others alongside their rights as individuals. Before we can move towards a more integrated ethical system for clinical care and research, however, we must acknowledge the very real legal barriers that have been enacted under the 20th century’s bioethics paradigm in the form of legislation like HIPAA’s Privacy Rule. As we move further into the Twenty-first century, the questions raised by traceback testing will challenge us to consider how to make genetic health information more accessible to those who stand to benefit from it the most: individuals and their families. A new bioethics paradigm that sees us all as sharing some level of individual responsibility towards the health and wellbeing of others through our genetic information is a provocative idea that deserves further discussion and analysis. In order to realize this shift, we may need to establish new legal and ethical pathways for traceback testing from millions of patient records and archived biological samples to the testing and care of those at-risk individuals who will benefit from it most.^90^

## Note

The authors have no conflicts to disclose.
